# Spatial profiles of markers of glycolysis, mitochondria, and proton pumps in a rat glioma suggest coordinated programming for proliferation

**DOI:** 10.1186/s13104-015-1191-z

**Published:** 2015-06-02

**Authors:** Emmanuelle Grillon, Régine Farion, Moshe Reuveni, Andrew Glidle, Chantal Rémy, Jonathan A. Coles

**Affiliations:** Université Grenoble Alpes, IRMaGe, 3800 Grenoble, France; Inserm, US 17, 3800 Grenoble, France; CNRS, UMS 3552, 3800 Grenoble, France; CHU de Grenoble, Hopital Michallon, IRMaGe, 3800 Grenoble, France; Institute of Plant Sciences, The Volcan Center, Bet Dagan, Israel; Department of Engineering, University of Glasgow, Glasgow, UK; Inserm, U 836, 3800 Grenoble, France; Institute of Infection, Immunity and Inflammation, University of Glasgow, 120 University Place, Glasgow, G12 8TA UK

**Keywords:** Glioblastoma, Glycolysis, Mitochondria, V-ATPase, Spatial structure, C6 glioma, Rat, GAPDH, Tom20, Immunofluorescence

## Abstract

**Background:**

In cancer cells in vitro, the glycolytic pathway and the mitochondrial tricarboxylic acid (TCA) cycle are programmed to produce more precursor molecules, and relatively less ATP, than in differentiated cells. We address the questions of whether and where these changes occur in vivo in glioblastomas grown from C6 cells in rat brain. These gliomas show some spatial organization, notably in the upregulation of membrane proton transporters near the rim.

**Results:**

We immunolabeled pairs of proteins (as well as DNA) on sections of rat brains containing gliomas, measured the profiles of fluorescence intensity on strips 200 µm wide and at least 3 mm long running perpendicular to the tumor rim, and expressed the intensity in the glioma relative to that outside. On averaged profiles, labeling of a marker of the glycolytic pathway, glyceraldehyde 3-phosphate dehydrogenase (GAPDH), was, as expected, greater in the glioma. Over distances up to 2.5 mm into the glioma, expression of a marker of the TCA cycle, Tom20, a pre-protein receptor on the translocation complex of the mitochondrial outer membrane, was also upregulated. The ratio of upregulation of Tom20 to upregulation of GAPDH was, on average, slightly greater than one. Near the rim (0.4–0.8 mm), GAPDH was expressed less and there was a peak in the mean ratio of 1.16, SEM = 0.001, N = 16 pairs of profiles. An antibody to V-ATPase, which, by pumping protons into vacuoles contributes to cell growth, also indicated upregulation by about 40%. When compared directly with GAPDH, upregulation of V-ATPase was only 0.764, SD = 0.016 of GAPDH upregulation.

**Conclusions:**

Although there was considerable variation between individual measured profiles, on average, markers of the glycolytic pathway, of mitochondria, and of cell proliferation showed coherent upregulation in C6 gliomas. There is a zone, close to the rim, where mitochondrial presence is upregulated more than the glycolytic pathway, in agreement with earlier suggestions that lactate is taken up by cells near the rim.

**Electronic supplementary material:**

The online version of this article (doi:10.1186/s13104-015-1191-z) contains supplementary material, which is available to authorized users.

## Background

Glioblastomas, which derive from the astrocyte lineage and are almost invariably fatal, are among that majority of cancers initiated by stochastic errors of DNA replication and not caused by genetic predisposition or environmental carcinogens [1]. Surgical resection, conventional radiation therapy and anti-angiogenic chemotherapy [2] have had only limited success in their treatment. Hence new therapies are required and their development might benefit from a better understanding of the physiology. Although there is considerable variety in the molecular phenotypes of human glioblastomas [3], the more malignant ones colonize the brain by growth of the tumor mass and by migration of individual cells away from the tumor rim [4–7], so we focus here on the rim.

The tumor grown in Wistar rat brain from the C6 cell line of transformed rat astrocytes colonizes the brain in a manner similar to many human glioblastomas [8–10]. By analyzing the distribution of antibodies to marker molecules on tissue sections, we have previously shown that the rim of a C6 glioma shows a degree of organization that is statistically significant when several spatial profiles are averaged. Notably, expression of the membrane Na^+^/H^+^ exchanger, NHE1 (SLC9A12), which contributes to cell migration by extruding protons [11–17] and interacting with the cytoskeleton [12, 18], is upregulated in a peak at the rim [19]. The internal H^+^ binding site of NHE1 is modified in cancer cells so that NHE1 continues to export protons even when the intracellular pH (pHi) rises higher than in normal cells [11, 12, 20–23]. The exported protons contribute to lowering extracellular pH (pHe) below the normal 7.3, and this acidic pHe assists invasion of host tissue by killing differentiated cells [24] and by activating metallo matrix proteases, which break down extracellular matrix and facilitate cell migration into host tissue [12, 25, 26].

The export of protons on NHE1 is intimately dependent on energy metabolism, since Na^+^/H^+^ exchangers require an inward gradient of [Na^+^], which is maintained by the ATP-consuming Na^+^ pump (or Na^+^-K^+^ ATPase). In differentiated cells, nearly all the ATP is produced by oxidative phosphorylation associated with the TCA cycle in mitochondria, the mitochondria being fuelled by pyruvate produced by the Embden-Meyerhof glycolytic pathway. In cancer cells, and other proliferating cells in culture, the glycolytic pathway and the TCA cycle are reprogrammed to produce intermediates necessary for the synthesis of the macromolecules of cell growth, rather than a maximum of ATP [27–31]. As part of this reprogramming, much of the pyruvate is converted to lactate, and tumors show a net excretion of lactate [32–34]. Lactate export (and import) is mediated by members of the monocarboyxlate transporter (MCT) family. In some tumors, including C6 gliomas, expression of the lactate transporter MCT1 is upregulated near the tumor rim [19, 35] an arrangement which has led to the hypothesis that some of the lactate produced deeper in a tumor diffuses towards the rim where it is taken up and oxidized [19, 35–37]. If this is true, then the ratio of oxidative phosphorylation to glycolysis in the rim might be greater than in the bulk of the tumor, and one aim of the present work is to see if there is evidence for this.

Also involved in both cell metabolism and the creation of an acidic pHe is the vacuolar H^+^-ATPase (V-ATPase), a proton pump. In addition to being a consumer of ATP [38–40], V-ATPase contributes to the metabolism of proliferating cells by transporting H^+^ into vacuoles (including endosomes, lysosomes and the Golgi apparatus) that are the sites of synthesis and degradation of macromolecules [39–42]. Upregulation of V-ATPase in cancer cell lines is associated with increased invasiveness [40, 43, 44] and V-ATPase has repeatedly been proposed as a target for cancer therapy [45–50]. In C6 cells, it is present on the plasma membrane [51] as well as vacuoles [43, 52]. It would be interesting to know how upregulated expression of V-ATPase compares with expression of the Embdem-Meyerhof pathway and this is the second question we address.

Nearly all of the extensive work on cancer metabolism and signaling has been done on cells in culture (see, e.g. [30, 53–55]); our aim here is to complement this with a description of spatial organization in a tumor. The mean distances from the rim to the peaks of NHE1 and MCT1 in C6 gliomas have been measured as 0.33 and 1.05 mm, respectively [19]. To obtain comparable spatial profiles (with resolution <10 µm over distances of millimeters), we tile-scanned tissue sections labeled with antibodies to marker molecules, and measured intensity profiles along strips perpendicular to the glioma rim, as previously [19]. Intensities were normalized with respect to the host tissue and a number of profiles from different sections were aligned and averaged (see “Methods”). The choice of markers was more limited than for cells in culture (for example, the mitochondrial label MitoTracker does not work on dead tissue), and we concentrated on obtaining statistically significant results for three markers. We used antibodies against glyceraldehyde 3-phosphate dehydrogenase (GAPDH) as a marker of the glycolytic pathway, Tom20, a receptor for pre-proteins that forms part of the translocase complex of the outer mitochondrial membrane [56, 57], and V-ATPase.

## Methods

### Ethics statement

All procedures involving animals conformed to European Council Directive 2010/63/UE and the study was approved by the Ethical Committee of the Grenoble-Institut des Neurosciences, agreement ID 004. Facilities for animal housing and procedures were approved by the French Ministry of Agriculture, licence B 38 516 10008 and all experimenters held personal licenses. The rats were sacrificed before the appearance of marked clinical symptoms.

### Preparation of the tumor model

C6 cells [8, 10] from the American Type Culture Collection were grown in DMEM containing 25 mM glucose and 2 mM l-glutamine (product 31966-021 from Invitrogen, Cergy Pontoise, France) to which was added 10% FBS (Invitrogen) and antibiotics. The rat glioma model was prepared as described [58]: male Wistar rats (200–230 g) were anesthetized with isoflurane and 10^5^ C6 cells in DMEM were injected stereotaxically in the right caudate nucleus. The growth of the tumor was monitored by MRI under isofluorane anesthesia at 4.7 T (Avance III console; Bruker, Grenoble MRI Facility IRMaGe) using a T2-weighted sequence (TR/TE = 4,000/33 ms, with repetition time 4,000 ms, and echo time 33 ms [59]). When the tumor diameter was 5–7 mm (20–25 days after implantation), the rat was decapitated, the brain was rapidly removed and frozen in isopentane at −80°C, and 10 µm coronal cryosections were cut at −20°C.

### Antibodies

Antibody against a conserved peptide of the E subunit of V-ATPase (SVSAEEEFNIEKLQLVEAEKKKIRQ) was prepared by Genemed Synthesis Inc. CA, USA and used at 1/500. This antibody labels V-ATPases in plants [60] and rat endothelial cells [61]. The antibody for GAPDH was a goat polyclonal NB300-320 from Novus Biologicals, used at 1/500. The antibody for Tom20 was a mouse monoclonal (Novus Biologicals H00009804_M01; called TOMM20) raised against a 146 AA recombinant protein, and used at 1/500. Immunolabeling of Tom20 has been shown to colocalize with the outer membranes of mitochondria in fish embryos [62].

Secondary antibodies were anti rabbit Alexa 488 or 633, anti-mouse Alexa 488, or 568, and anti-goat Alexa 546, all at 1/500 and from Invitrogen.

### Immunofluorescence labeling

The sections were fixed for 10 min in 4% paraformaldehyde, washed and incubated for 1 h in 3% BSA at room temperature, then incubated with a pair of first antibodies in 3% BSA for 16 h at 4°C. After three rinses in PBS, the secondary antibodies were applied for 1 h at room temperature. After three more rinses, the sections were mounted in GelMount (MM, France) containing bisbenzimide trichlorohydride (Hoechst 33342, 1 µg/ml).

### Imaging

Fluorescence labeled sections were observed on a Zeiss LSM 510 META. Tumors were initially located by bisbenzamide fluorescence using a xenon lamp and full field illumination. Tile scans were made with a ×10 EC Plan-Neofluor objective over areas that included a 200 µm wide strip perpendicular to the tumor rim (e.g., 1 × 6 or 3 × 5 frames). Each frame was 512 × 512 pixels and intensity was coded at 8 bits. Each tile scan was opened in ImageJ and, if necessary, the image was rotated, so that a border, well-defined by bisbenzamide, was approximately vertical, the tumor being to the right. A horizontal strip 200 µm wide and 4–5 mm long covering intra- and extratumoral tissue was selected. On the tile scan image of the second antibody, the same strip was selected in ImageJ by reference to the coordinates. The “Plot Profile” function was applied and the listed values pasted in GraphPad Prism. For each strip, the edge of the tumor was identified on the graph of the bisbenzamide labeling and the x-scale shifted so that the tumor edge was defined as x = 0. Intensity values over 1 mm or more outside the tumor were selected, pasted in a new project, and averaged. These baseline values were then used to normalize the complete profiles. The abscissae of all the profiles for each antibody were then aligned, and the profiles averaged. Ratios were calculated in Excel (Additional files 1, 2).

### Statistics

Significance was calculated with Student’s *t* test. No outliers were excluded.

## Results

### GAPDH and Tom20 are upregulated throughout C6 gliomas

**Figure 1:**
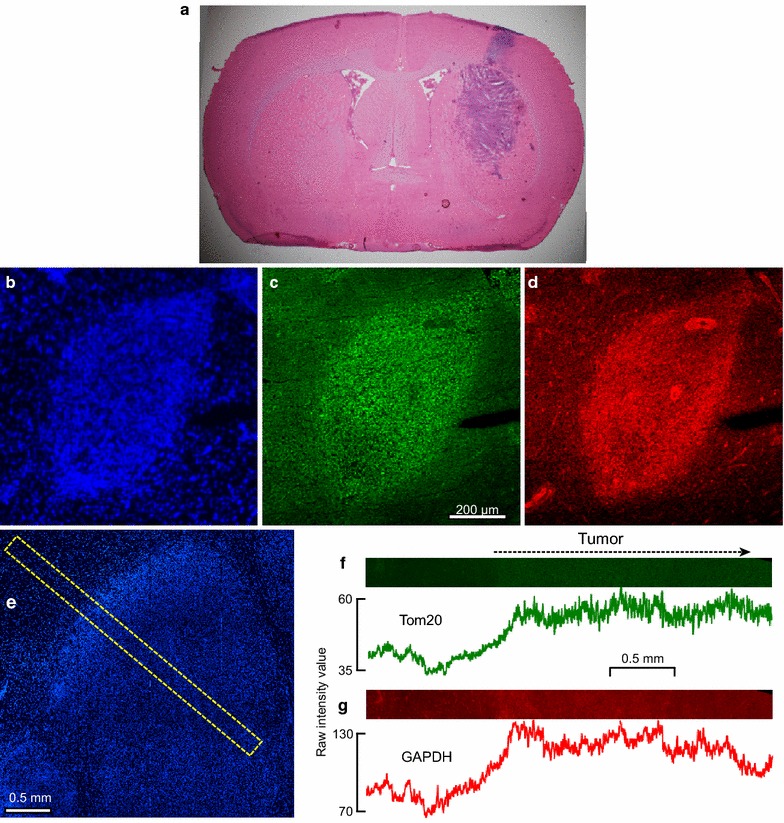
Upregulation of GAPDH and Tom20 in C6 gliomas. **a** A section of rat brain stained with hematoxilin and eosin to show a glioma produced by implantation of C6 cells in the right caudate nucleus. In this case, cells also grew in the region of the syringe needle track through the cortex. (**b**–**d**). A small glioma labeled by bisbenzamide (**b**) and with antibodies against Tom20 (**c**) and GAPDH (**d**). The brightness and contrast have been increased. **e** Illustration of a strip perpendicular to the rim of a glioma (labeled with bisbenzamide) and along which intensity profiles were measured. **f**, **g** Tom20 and GAPDH fluorescence along such a strip (barely visible without enhancement). The graphs show the raw intensity values given by ImageJ.

A section of a rat brain containing a C6 glioma stained with hematoxilin-eosin is shown in Figure 1a. Immunolabeled gliomas were visualized by fluorescence from bisbenzamide (Hoechst 33342) labeling (Figure 1b, e). Labeling of both GAPDH and Tom20 was detected outside tumors, and labeling was more intense within tumors. This is illustrated qualitatively in a section of a small tumor shown in Figure 1b–d with brightness and contrast enhanced. At higher magnification, GAPDH appeared to be localized near the peripheries of cells, while Tom20 was concentrated in small objects that could be mitochondria (Additional file 3). To determine profiles of mean upregulation across the tumor rim in several brain sections, tile scans were made and radial strips selected (Figure 1e). In unenhanced images, fluorescence was often barely perceptible to the eye, but upregulation within the tumor was apparent on intensity profiles (Figure 1f, g).

**Figure 2:**
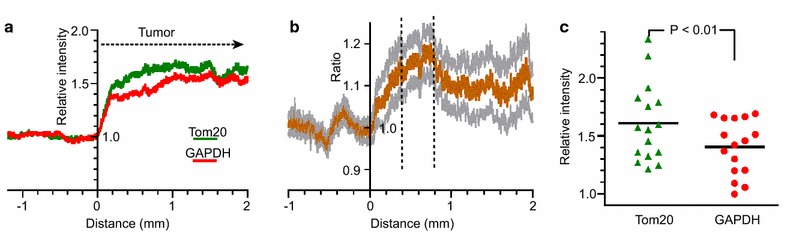
Averaged profiles of upreglation of Tom20 and GAPDH. **a** Means of 16 pairs of profiles for Tom20 and GAPDH. **b** The mean ratios of individual pairs of profiles, Tom20/GAPDH. The ratio is greatest in the range 0.4–0.8 mm (*vertical lines*). **c** Mean values for individual profiles over the distance 0.4–0.8 mm into the glioma.

The averages of 16 pairs of profiles are shown in Figure 2a where it is seen that labeling of both GAPDH and Tom20 increased rapidly over the first 0.2 mm into the tumors. The mean intensity relative to outside the tumor over the range 1.5–2.0 mm into the tumor was calculated for each of the 16 profiles. Over this distance, the mean upregulation of GAPDH was 1.57, SD = 0.32, N = 16, and of Tom20, 1.50 ± 0.44. The upregulation of both GAPDH and Tom20 was significant with *p* < 0.0001 and *p* = 0.0004 respectively. Mean upregulation of Tom20 from 0.2 to 2 mm was generally slightly greater than that of GAPDH. To examine this, the ratios of the upregulation Tom20/GAPDH were calculated for each pair of profiles (Additional file 1), and the mean and SEM plotted (Figure 2b). The greatest difference was over the range ≈ 0.4–0.8 mm (dashed lines in Figure 2b) where the mean ratio was 1.159, SEM = 0.001, N = 16 pairs of profiles, which is significantly greater than 1 with *p* < 0.0001. Even deeper into the tumor (2.0–2.5 mm) the mean increase in Tom20 labeling was statistically greater that of GAPDH, the mean ratio over 2.0–2.5 mm being 1.084 (SEM. 0.001, N = 11 profiles, *p* < 0.0001). These results suggest that in this sample of gliomas mitochondrial presence was, on average, upregulated slightly more than the glycolytic pathway up to at least 2.5 mm from the rim, with a peak in the ratio near the rim. There was, however, considerable variation from one profile to another (Figure 2c).

### V-ATPase upregulation

**Figure 3:**
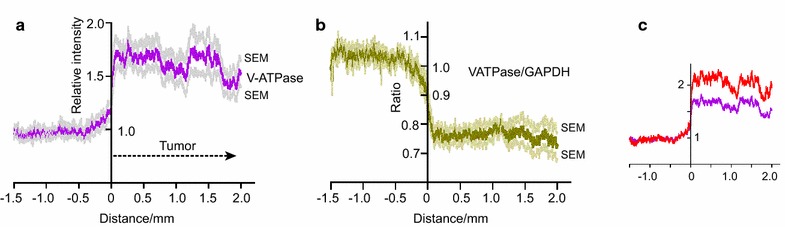
V-ATPase upregulation. **a** Average of 9 profiles of VATPase with SEM for each data point. N = 9. **b** The ratio of VATPase upregulation to GAPDH upregulation was calculated for each profile and averaged. **c** The double labeling for V-ATPase and GAPDH used for (**a**, **b**) was from a glioma different from those used for Figures 1, 2, and it had greater upregulation of GAPDH (*red*).

Upregulated glycolysis and mitochondrial activity suggest cell proliferation, for which acidic intracellular vacuoles are required. As expected, labeling of V-ATPase was upregulated within the gliomas (Figure 3a). When the intensity relative to outside over the distance 0.5–2.0 mm into the glioma was calculated for each profile, the mean value for the nine profiles was 1.61, SD = 0.34, *p* < 0.0001. GAPDH was labelled on the same sections and the ratio of V-ATPase upregulation to GAPDH upregulation for each pair of profiles was calculated and averaged (Additional File 2). V-ATPase was upregulated less than GAPDH, the mean ratio over 0.5–2.0 mm being 0.764, SD = 0.016, *p* < 0.0001 (Figure 2b). The sections were from gliomas different from those of Figure 2; the upregulation of GAPDH was greater and was not compared directly with upregulation of Tom20 (Figure 3c).

## Discussion

Antibody labeling of GAPDH, Tom20 and V-ATPase was, on average, markedly increased relative to labeling outside the C6 gliomas, an increased level extending from the tumor rim to at least 2.5 mm into the tumor. This pattern is very different from that shown previously for NHE1, which is upregulated in a peak at the rim, and for the carbonic anhydrase, CAIX, which is not upregulated in this zone [19]. These radical differences appear to rule out the possibility that the observed profiles of antibody labeling are the result of some artifactual tumor-specific increase in the efficacy of antibody binding, or a reduction in the extracellular space fraction, rather than a true increase in protein expression.

There was considerable variation in the measured intensity of the labeling (always expressed relative to the intensity outside the glioma) from one profile to another and one glioma to another. For example, when the mean value for each profile over the distance 0.5–1 mm was calculated for the series of sections for which both GAPDH and V-ATPase were labeled, the coefficients of variation (SD/mean) for the 9 pairs of profiles were 0.229 for V-ATPase and 0.251 for GAPDH. A possible artifactual contributor to this variance is tissue distortion caused by stretching or compression of parts of sections. When the corresponding ratio V-ATPase/GAPDH was calculated, its coefficient of variation was much less, being 0.103. i.e., the ratio of expression of V-ATPase relative to expression of GAPDH is quite tightly controlled. Inspection of Figure 2b, c suggests that the same is true of Tom20 and GAPDH. These results strongly suggest that the different components of cell activity are well-coordinated in C6 gliomas. An unknown factor is how tightly the expression of GAPDH, Tom20 and V-ATPase are regulated in normal brain.

### Upregulation of Tom20

The production of lactate by tumors has sometimes obscured the fact that oxygen consumption is, in most tumors, greater than in normal tissue [34, 63]. In rat gliomas, the oxygen saturation in much (or all) of the volume is as high as in normal brain tissue [64], although zones of hypoxic necrosis can develop [65]. In keeping with this expected availability of oxygen in C6 gliomas, expression of CAIX, which is upregulated by Hypoxia Inducible Factor 1 alpha (HIF1alpha; [66]) has been found not to be upregulated over at least 2 mm in from the rim [19]. It is therefore unsurprising that Tom20, a component of mitochondria, should be present, as we find.

### Comparison of Tom20 and GAPDH

The metabolic pathways associated with glycolysis and with mitochondria can be programmed in either of two directions, to produce a maximum of ATP, or to produce precursor molecules for cell growth and proliferation, the latter function becoming predominant in proliferating cells [28, 67]. That both Tom20 and GAPDH were upregulated with a ratio close to 1 is reminiscent of the finding by Gullino et al. [34], in various types of tumor, that “glucose consumption and lactate elimination were in direct proportion to the oxygen utilized and a lack of oxygen blocked both of them”.

Although the upregulation of Tom20 compared to upregulation of GAPDH was greatest near the rim, Tom20 upregulation was still significantly higher than GAPDH deeper (>1 mm) into the tumor, although only by <10% (Figure 2b). Since C6 gliomas release lactate, there is presumably less pyruvate available to feed into the TCA cycle. Instead, part of the upregulation of Tom20 may be necessary to metabolize glutamine, supplied by the blood and a major entrant of the TCA cycle in cancer cells [27, 68–70].

**Figure 4:**
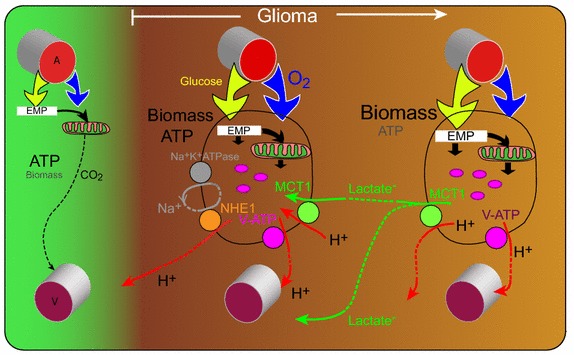
Scheme summarizing the results and discussion. Compared to non-tumoral tissue, the tumor takes up more glucose (*yellow arrows*) and oxygen (*blue arrows*). Part of the mitochondrial metabolism in the rim is fuelled by lactate. NHE1 has a specific role in migrating cells. V-ATPase is present both on plasma membranes and intracellular vacuoles (*pink*). *EMP* Embden-Meyerhof Pathway.

Closer to the rim (<0.8 mm), on sections in which Tom20 was compared directly with GAPDH, GAPDH expression was somewhat reduced so that there was a marked peak in the ratio Tom20/GAPDH. The peripheral location of MCT1 in cervix squamous carcinoma has led to the hypothesis that there can be net production of lactate within a tumor and that some of this is taken up close to the rim as a fuel for oxidative phosphorylation [35, 36]. A peak in MCT1 near the rim has also been reported in C6 gliomas [19]. Transfer of lactate, or other metabolic fuel, from one cell to another, is well known as a physiological phenomenon in muscle and nervous tissue [71–74]. The observed excess of Tom20 over GAPDH near the rim seems to fit well with the hypothesis of lactate transfer in C6 gliomas (Figure 4).

### V-ATPase

The presence of V-ATPase has been reported in several types of tumor where it subserves the handling of molecules for cell growth in intracellular vacuoles, and also contributes to the export of protons across the plasma membrane [40, 43, 44, 51, 52]. For the C6 gliomas of Figure 3a, the mean upregulation of V-ATPase expression was about 1.7 compared to normal brain, and it was tightly regulated against GAPDH (Figure 3b). In these sections, expression of GAPDH was upregulated more than V-ATPase. A possible explanation is that upregulation of glycolysis in C6 gliomas serves two functions: to support an increase in production of biomass (which requires V-ATPase) and also to produce pyruvate for ATP production (which does not require V-ATPase).

## Conclusions

The upregulation of expression of V-ATPase and markers of glycolysis and mitochondria illustrate the co-ordinated programming of these engines of anabolism. This coordination extends to some stochastic spatial organization supporting the prediction that some of the fuel for oxidative phosphorylation in the tumor rim is supplied by glycolysis deeper into the tumor. The relatively simple technique we have used has obvious extensions to questions including transmembrane transport of glutamine [68, 69] and HCO_3_^−^ [75].

## Availability of supporting data

The data sets supporting the results of this article are included within the article and its additional files.

## Additional files

**Additional file 1:** Numerical tabulation of profiles of Tom20 and GAPDH after normalization and alignment
**Additional file 2:** Numerical tabulation of profiles of V-ATPase and GAPDH after normalization and alignment
**Additional file 3:** Microscopic localization of GAPDH, Tom 20, and V-ATPase

## Abbreviations

CAIX: carbonic anhydrase IX
DMEM: Dulbecco’s modified Eagle’s medium
EMP: Embden-Meyerhof pathway
GAPDH: glyceraldehyde 3-phosphate dehydrogenase
MCT1: monocarboxylate cotransporter 1 (SLC16A1)
NHE1: sodium hydrogen exchanger 1 (SLC9A12)
Tom20: translocase of the mitochondrial outer membrane 20
V-ATPase: vacuolar H^+^-ATPase
