# Supplementary material for: Spatial profiles of markers of glycolysis, mitochondria, and proton pumps in a rat glioma suggest coordinated programming for proliferation
Source: BMC Res Notes. 2015 Jun 2;8:207. doi: 10.1186/s13104-015-1191-z (PMC4467611; doi:10.1186/s13104-015-1191-z)
Supplement: Supplementary file 3 — Additional file 3: Microscopic localization of GAPDH, Tom 20, and V-ATPase [file 13104_2015_1191_MOESM3_ESM.docx]

### **Microscopic localization of GAPDH , Tom 20, and V-ATPase.**

### Figs 1 and 2 in the main text show that on a scale of hundreds of microns, increased labeling of the proteins GAPDH, Tom20 and V-ATPase in the tumors was broadly similar in that it rose sharply at the rim to a plateau that was maintained to at least 2 mm into the tumor. To see how these proteins were distributed at a cellular level, we made additional images at higher magnification.

### In raw images, differences between Tom20 (Additional Fig. 1A) and GAPDH (Fig. 1B) were not striking. But subtraction of one from the other revealed differences. Tom20 labeling predominated in small objects, 2-3 µm across, which may be mitochondria (Fig. 1C). In contrast, GAPDH predominated closer to the cell membrane [1], including areas where no Tom20 predominance was seen (Fig. 1D, arrows).

###
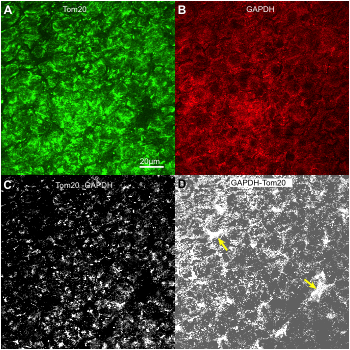


### **Figure 1. Localization of GAPDH compared to Tom20 in a tumor.** **A.**Tom20. **B.** GAPDH. **C**. Image (A) - Image (B). **D**. Image (B) - Image (D). After the image subtractions, images were brightened and contrast increased

###

### At intermediate magnification, V-ATPase labeling was intense close to blood vessels, and so was Tom20 (Fig. 2A,B). However, image subtraction showed that the V-ATPase labeling predominated closer to the lumen while Tom20 predominated slightly further away (Fig. 2C,D). At higher magnification, very little difference was seen between V-ATPase and GAPDH outside a tumor (Fig. 2E-G), but within the tumor, V-ATPase predominated in small spots, presumably vesicles (Fig. 2H-J).


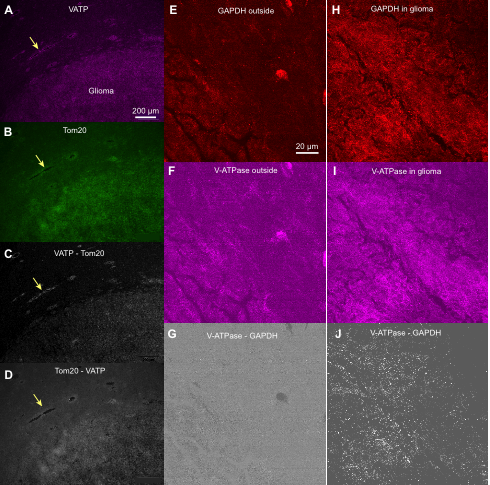


**Figure 2. Localization of V-ATPase compared to GAPDH and Tom 20**. (**A-D**). Low magnification images showing part of a tumor. **A.** VATPase, **B**. Tom20. **C.** Image (A) - Image (B). **D.** Image (B) - Image (A). The arrows indicate a blood vessel. **E-G.** Higher magnification, outside a tumor.  **E.** GAPDH. **F**. V-ATPase. **G** Image (F) - Image(E). **H-J.** Within a tumor. **H**. GAPDH. **I.** V-ATPase. **J**. Image (I) - Image (H). After the image subtractions, images were brightened and contrast increased.

**Reference**

1. Epstein T, Xu L, Gillies RJ, Gatenby RA. Separation of metabolic supply and demand: aerobic glycolysis as a normal physiological response to fluctuating energetic demands in the membrane. Cancer & Metabolism. 2104; 2: 7.
